# Porous Carbon Nanofoam Derived From Pitch as Solar Receiver for Efficient Solar Steam Generation

**DOI:** 10.1002/gch2.201900098

**Published:** 2020-02-20

**Authors:** Lihua Chen, Shujing Zhao, Qi‐Meige Hasi, Xiaofang Luo, Chuantao Zhang, Hailing Li, An Li

**Affiliations:** ^1^ College of Chemical Engineering Northwest Minzu University Key Laboratory for Utility of Environment‐Friendly Composite Materials and Biomass in University of Gansu Province Lanzhou Gansu 730030 P. R. China; ^2^ Department of Chemical Engineering College of Petrochemical Engineering Lanzhou University of Technology Lanzhou 730050 P. R. China

**Keywords:** conversion efficiency, pitch, porous carbon, solar steam generation

## Abstract

Photothermal‐material‐assisted solar‐steam generation has recently attracted intensive attention due to its superior evaporation rate with high energy conversion efficiency for desalination. In this work, a simple approach for fabrication of porous carbon nanofoam (PCN) is reported, which is prepared by the carbonization of pitch using a combination of CaCO_3_ and NaCl templates, Meanwhile, NaCl saturated solution acts as a porogen to produce micropores and mesopores as solar receiver for efficient solar steam generation. The as‐prepared PCN shows excellent porosity and mesoporous feature with an average pore size of 26.8 nm. It also shows superior light absorption of 88% and better thermal insulation (thermal conductivity 0.993 W m^−1^ K^−1^). Based on these characteristics, the as‐prepared PCN can be used as a promising solar receiver. Under 1 sun, 2 sun, and 3 sun irradiation, the PCN‐based solar receiver shows high energy conversion efficiencies of 88%, 86%, and 84%, respectively. Taking advantage of the abundant, low‐cost, and commercial availability of pitch as well as its simple and cost‐effective manufacture method, the PCN‐based solar receiver may hold great potential for a broad variety of solar‐steam generation applications, for instance, fresh water production, power generation, desalination, and so on.

## Graphical abstract

Porous carbon nanofoam derived from the pyrolysis of a CaCO_3_ nanoparticle/pitch mixture is first prepared as a novel photothermal material, which shows a high evaporation efficiency of 88% under 1 kW m^−2^ illumination, thus showing great potential for practical solar steam generation.

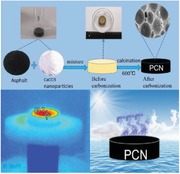

## 1 Introduction

The severe issues of the scarcity of water resources or water pollution have seriously restricted the sustainable development of modern society.^[^ 1 ^]^ Therefore, the exploitation of high‐performance and cost‐efficient strategy for production of fresh water is urgently needed. Though various modern technologies, for example, microfiltration,^[^ 2 ^]^ ultrafiltration^[^ 3 ^]^ reverse osmosis,^[^ 4 ^]^ multi‐effect distillation,^[^ 5 ^]^ multi‐stage flash,^[^ 6 ^]^ and adsorption,^[^ 7 ^]^ have been employed for desalination to this end, usually the need of huge energy consumption makes them unsuitable for large‐scale applications, especially in those un‐developed regions or countries.

Recently, solar steam generation, which employs green and inexhaustible solar energy as energy input mediated by solar receivers, has emerged as one of the cutting‐edge technologies with great potentials for a wide range of applications such as wastewater treatment, fresh water production, power generation, desalination, and so on.^[^ 8, 9, 10, 11, 12, 13, 14 ^]^ The most impressive feature of solar steam generation is its high energy conversion efficiency,^[^ 15, 16, 17, 18, 19, 20 ^]^ which is benefited from its unique “interfacial‐evaporation” manner, by comparison with the traditional bulk water vaporization. According to these design principles, a wide range of photothermal materials have been created so far, including carbon‐based materials,^[^ 21, 22, 23 ^]^ metallic nano‐particles,^[^ 24, 25, 26 ^]^ natural materials,^[^ 27, 28 ^]^ inorganic silica‐based materials,^[^ 29 ^]^ and synthetic porous polymers.^[^ 30, 31 ^]^ For example, plasmonics have quite extensive applications in the area of solar energy conversion as the local plasmon resonance on the surface of metal nanoparticles results in a significant increase in magnetic field strength, and thus localization of heat around the nanoparticles,^[^ 32, 33, 34, 35, 36 ^]^ the method of generating steam require high light intensities (usually above 10 kW m^−2^), which gets a relative low vapor generation efficiency (usually below 70%) and increases the surface heat loss. To improve the light absorption ability of a solar receiver, the common approach to this end is to create a light absorption layer^[^ 37, 38 ^]^ (usually use black materials such as carbon or polymers, e.g., polypyrrole) on the surface, a porous layer with better thermal insulation. Along this line, a number of solar receivers are derived from natural products,^[^ 39 ^]^ polymers,^[^ 40 ^]^ clay, or composites^[^ 41 ^]^ and so on. Alternatively, carbon aerogel materials (such as carbon nanotube aerogels and graphene aerogels) have a highly developed pore structure, low thermal conductivity, and a wide range of light absorption, making them potential solar photothermal materials for desalination.^[^ 42 ^]^ Compared with those double‐layered solar receiver usually suffering the drawbacks of multi‐step or complicated process, carbon aerogel materials themselves have excellent light absorption due to their carbon in nature, thus showing advantage of simple fabrication. On the other hand, rich resources of carbon materials or their precursors with low‐cost make them promising candidates as solar photothermal materials especially for practical applications

Inspired by the previous works reported by others and us,^[^ 43, 44, 45, 46 ^]^ in this work, we demonstrate a new approach for facile fabrication of porous carbon nanofoam (PCN) as efficient solar receiver for solar steam generation. The as‐resulted PCN has good thermal stability, excellent light absorption, low apparent density, and thermal conductivity, which render it an ideal candidate as a solar photothermal material. Based on these merits mentioned above, the PCN shows superior solar photothermal performance with a high evaporation rate of 1.68 kg m^−2^ h^−1^ achieved under 1 sun irradiation. The solar energy conversion efficiency was measured to be as high as 88%, 86%, and 84% under 1 sun, 2 sun, and 3 sun irradiation, respectively. Taking advantages of their high solar steam generation performance, low‐cost raw chemicals, and simple manufacture process, the PCN may have great potentials as a kind of promising solar receiver for solar steam generation.

## 2 Results and Discussion

**Figure 1:**
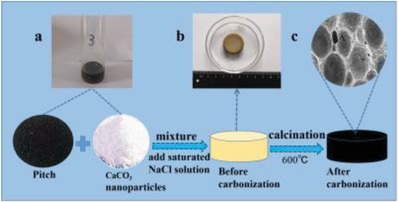
Preparation process of pitch/CaCO_3_ nanoparticle particles. a) Mixing the solution evenly. b) Photo of sample after molding. c) Partial electron microscope image of PCN.

A detailed procedure for preparing a CaCO_3_ nanoparticle/pitch mixture is shown in **Figure** **1**. First, the pitch was dissolved in methylene chloride as a solvent, then the pitch and CaCO_3_ nanoparticle was uniformly mixed at a ratio of 1:14, and a saturated NaCl solution of 10 mL was added thereto and stirring was continued. Thereafter, as shown in Figure 1a, the sample was naturally air dried at normal temperature. The prepared PCN has a diameter of about 2.5 cm (Figure 1b). Finally, carbonization was carried out in a tube furnace. The carbonization temperature was 600 °C, the carbonization time was 2 h, and the heating rate was 2 °C min^−1^. The color of the sample changed from brown to black, PCN was washed with 1 m HCl to remove CaCO_3_ nanoparticles. Partial electron microscope image of PCN finished products is shown in Figure 1c.

**Figure 2:**
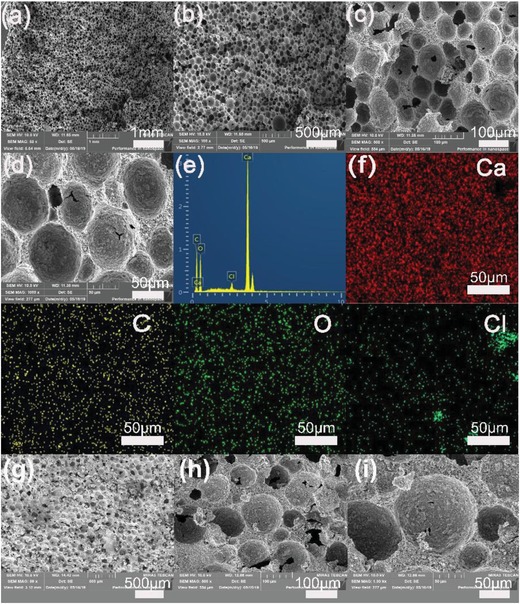
a) SEM images of 50 times PCN. b) SEM images of 100 times PCN. c) SEM images of 500 times PCN. d) SEM images of 1000 times PCN. e) EDS spectra for PCN area in. f) EDS mapping of PCN surface. Scale bar: 50 µm. g–i) A cross‐section SEM image of PCN.

As shown in **Figure** **2**d, the morphologies and structures of the PCN were characterized by scanning electron microscopy (SEM), which image showing a spherical macroporous skeleton with a diameter of about 50 µm, the roughly ordered micropores can be clearly seen at 1000×. Scanning electron microscope (SEM) images at 50× (Figure 2a), the particles formed by CaCO_3_ and pitch are roughly uniform and spherically clustered together to form roughly uniform micropore and mesopore arrangement. SEM showed that micropore diameters are about 100 µm, 5 nm, or bigger. As shown in Figure 2e, the elements of the PCN are mainly C, O, Cl, and Ca. To determine the distribution of individual elements mapping of them were further observed by energy dispersive spectroscopy (EDS). The results revealed the uniform distribution of the C, O, and Ca elements on the surface of PCN coating, uniform distribution is beneficial for light absorption. In addition, a small amount of Cl elements is present (Figure 2f). We can see in the figure (Figure 2g–i), a cross‐section SEM image of PCN, the pores being roughly uniform and tightly connected together, providing good transport for water molecules.

**Figure 3:**
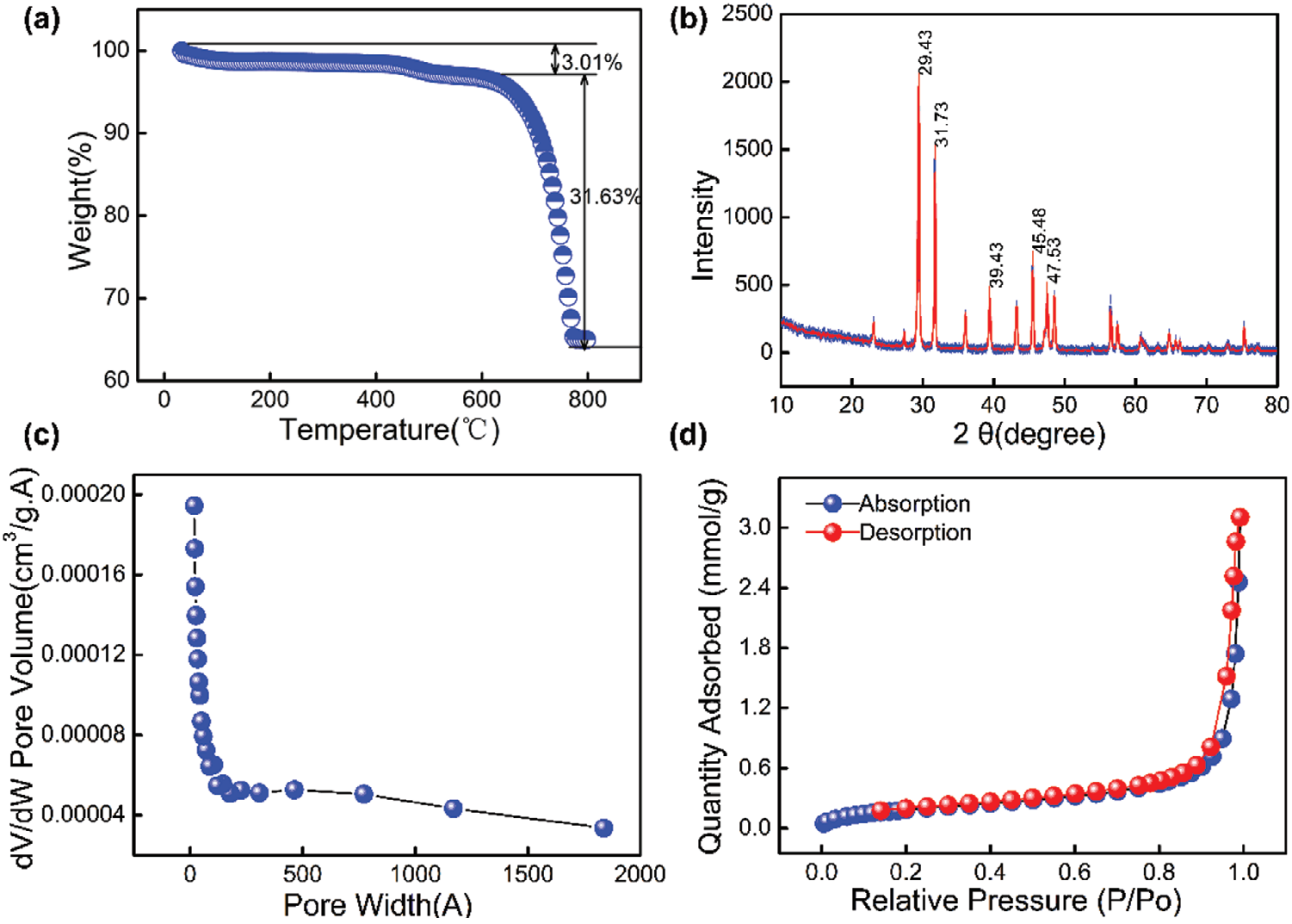
a) TGA of the PCN‐based composites. b) XRD patterns about the PCN. c) N_2_‐adsorption/desorption isotherms of PCN. d) BJH adsorption dV/dW pore volume.

Thermogravimetric analysis (TGA) was used to characterize and decompose. TGA was performed on the surface of pitch samples, and the results are shown in **Figure** **3**a; the decomposition of PCN is not obvious at (0–600 °C), and the mass loss is 3.01%, indicating that the thermal stability of PCN is relatively good. Significant decomposition occurred in the range of (600–800 °C), and the mass loss of prepared PCN was 31.53% in this temperature range. After 800 °C, the sample curve tends to be stable, indicating that PCN has been completely decomposed. The powder X‐ray diffraction (XRD) pattern is shown in Figure 3b, showing obvious characteristic peaks at 29.43°, 31.73°, 39.43°, 45.48°, and 47.53°, indicating the presence of crystals. CaO's diffraction peak is 29.43°; due to CaCO_3,_ nanoparticles in the sample were decomposed into CaO by heat, the crystal characteristic peak appeared. Saturated saline NaCl was also used in the experiment, which was also an ionic crystal, so the diffraction peak appeared. The specific surface area analysis of BET is shown in Figure 3c,d. We can see that N_2_‐adsorption/desorption measurement is 77.3 k, and the BET specific surface area of PCN is 16.0740 m^2^ g^−1^. The single‐point total pore volume of the pores at *P*/*P*_0_ = 0.9916 was found to be 0.1077 cm^3^ g^−1^ for PCN. Average pore width of adsorption (4 V A^−1^): 284.250 A; average pore width of desorption (4 V A^−1^): 225.361 A.

**Figure 4:**
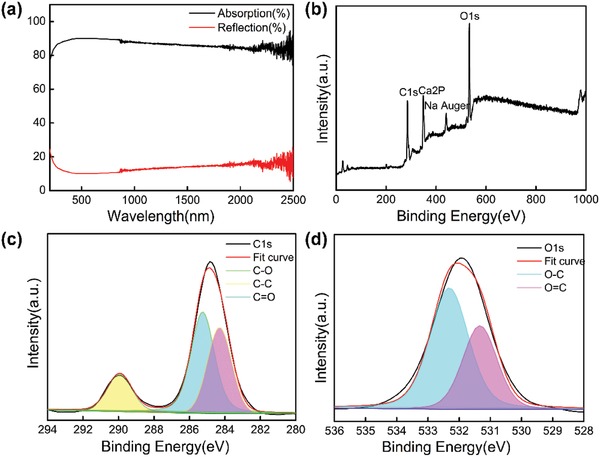
a) The light absorption of the PCN from 200 to 2500 nm. b) XPS spectrum of the PCN. c) C1s XPS spectra of the PCN. d) O2s XPS spectra of the PCN.

As shown in **Figure** **4**a, the absorption efficiency was then calculated by *A* = 1 − *R* − *T*, where *R* and *T* are the reflection and transmission efficiency, respectively. The optical absorption of the PCN studied using a UV‐3600 spectrophotometer exhibited excess 86% light absorption in a wide wavelength range from 200–2500 nm, indicating the excellent light absorption of the PCN. The X‐ray photoelectron spectroscopy (XPS) revealed that PCNs were largely composed of hydrophilic materials containing oxygen‐containing groups such as C=O and C—O (Figure 4b–d). 350 eV is the Ca2P peak and 450 eV is the Na auger peak. C1s (285 eV) spectra can be decomposed into three peaks, peaks C—O, C=O, and C=C, respectively. O1s spectra can be decomposed into two peaks; the peaks are O—C and O=C.

**Figure 5:**
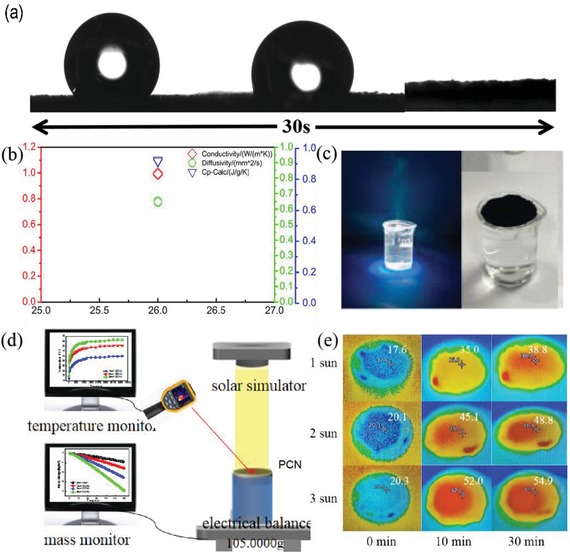
a) Image of water droplets immersed in PCN. b) Thermal conductivity, diffusion coefficient, and specific heat of PCN. c) Blank group in dark conditions. d) A schematic diagram of a solar drive system consisting of a solar stimulator, computer, electronic analytical balance, and an infrared camera. e) Infrared images under different sun illumination and time.

**Figure 6:**
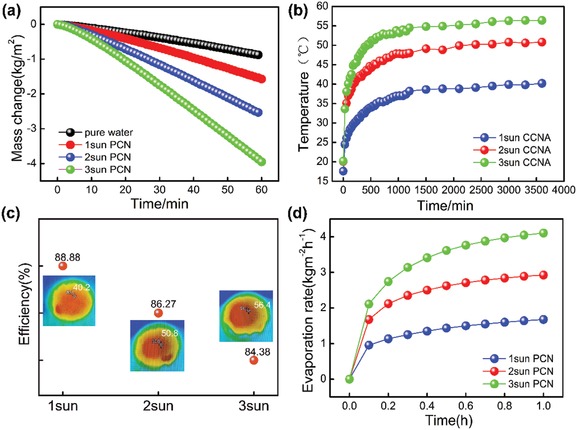
a) Mass change of water over time at an optical density of 1, 2, 3 kW m^−2^. b) The temperature of PCN under solar light irradiation as a function of time. c) Solar steam efficiency of PCN under different illumination. d) Time‐dependent evaporation rate of PCN under different illumination.

As shown in **Figure** **5**a, within 3s, the water drops were completely immersed into the surface of the sample; the data show that PCN is hydrophilic. As shown in Figure 5b, the thermal conductivity of one PCN sample at 26 °C was 0.992, 0.985, 1.001, and 0.993, respectively. The diffusion coefficients are 0.647, 0.643, 0.653, and 0.647, and the specific heat is 0.914, 0.915, 0.926, and 0.919, respectively. It can be seen from the above data that PCN is a low thermal conductivity material with good insulation performance.^[^ 47, 48, 49, 50 ^]^ In addition, taking inherent merits of the PCN composites such as unique porous monolithic structure and high chemical and thermal stability,^[^ 51 ^]^ it should be a promising candidate for the solar steam generator. The results, which are illustrated in **Figure** **6**a show that the amount of water evaporated increased with increasing solar radiation. The evaporation rates were calculated from the slope of the time‐dependent mass change curves. Figure 5c shows the blank group in dark conditions. Moreover, the solar steam generation experiment was conducted by using a lab‐made, real‐time measurement system (Figure 5d), which consisted of a solar simulator, computer, infrared camera, and electronic balance. Figure 6b shows that with increasing solar radiation, the surface temperature of the sample continues to increase. The surface temperature of PCN can reach 35 °C in 10 min, then reach a maximum temperature of 40.2 °C under 1 sun illumination, a maximum temperature of 50.8 °C under 2 sun illumination, and a maximum temperature of 56.4 °C under 3 sun illumination (Figure 5e). From Figure 6c, we can see the 1h energy efficiency measurement error of PCN under different solar illumination. According to the calculation of water evaporation efficiency formula, the efficiency at 1 sun is 88%, the efficiency at 2 sun is 86%, and the efficiency at 3 sun is 84%. This phenomenon is mainly attributed to considerable amounts of water that did not experience the phase transition (from liquid to vapor) to vapor into air during solar steam generation, which significantly saved energy and led to the increase of efficiency. In addition, based on this method, the PCN composite has a simple process, one‐shot molding, large scale, and is not subject to material size and shape restriction with great practical value for the app. The evaporation rate calculated from the slope of the time dependent mass change curves are shown in Figure 6d. The evaporation rate of PCN composites under 1 sun stimulation was 1.68 kg m^−2^ h^−1^; the evaporation rate under 2 sun stimulation was 2.92 kg m^−2^ h^−1^; and the evaporation rate under 3 sun was 4.10 kg m^−2^ h^−1^, indicating that the high evaporation rate of PCN should be attributed to its largest temperature rise, its excellent ability to convert light into thermal energy for efficient solar steam generation. Composite materials significantly increase the evaporation rate of water; it can be seen from the above that PCN has great practical application value.

## 3 Conclusions

In conclusion, we have demonstrated an approach for facile fabrication of a novel solar receiver, that is, PCN, which was prepared by the carbonization of pitch using the combination of CaCO_3_ and NaCl templates. The as‐resulted PCN shows superior light‐to‐heat conversion performance, for example, high solar energy conversion efficiencies of 88%, 86%, and 84% can be achieved under 1 sun, 2 sun, and 3 sun irradiation, respectively, which may be attributed to its comprehensive properties such as better thermal insulation, desired porous structure, and excellent light absorption. More interestingly, by taking advantage of the abundant, low‐cost, and commercial availability of pitch as well as its simple and cost‐effective manufacture method, such PCN‐based solar receiver may hold great potential for a broad variety of solar steam generation applications, for instance, wastewater treatment, fresh water production, power generation, desalination, and so on.

## 4 Experimental Section

##### Materials Preparation

Pitch was from China Petroleum Engineering Construction Co., Ltd. Nano‐CaCO_3_ particles were supplied by Beijing Dekedao Gold Technology Co., Ltd. Dichloromethane was supplied by Shanghai Maclean Biochemical Technology Co., Ltd. NaCl was from Guangdong Guanghua Sci‐Tech Ltd. No further purification were done of the reagents.

##### Experimental Details

In this study, pitch and nanometer CaCO_3_ were used as raw materials. Pitch (0.5 g) was added to dichloromethane (10 mL) and magnetically stirred for 20 min to completely dissolve it. Then nanometer CaCO_3_ powder (7 g) and saturated salt water (10 mL) were added and stirred evenly for 30 min. The sample was molded at room temperature; it was carbonized in an N_2_ protected tubular furnace. The carbonization temperature was 600 °C, the heating rate was 2 °C min^−1^, and the insulation time to obtain PCN was 2 h. Finally, PCN was washed with 1 m HCl to remove the CaCO_3_ nanoparticles.

##### Experimental Set‐Up for Steam Generation

The steam generation experiments were performed under a homemade optical system, with a solar simulator, and the temperature of the PCN surface was observed with the assistance of an IR thermal camera (Thermal Imager TESTO 869, Testo SE & Co. KGaA, Germany). The experiments were typically conducted at an ambient temperature of 20 °C and humidity of ≈40%. The PCN floated on the water with the help of foam; the mass change was measured by a high accuracy balance (0.0001 g in accuracy) and then real‐time communicated to a computer for the evaluation of the evaporation rate and solar‐thermal conversion efficiency.

##### Characterization

The morphologies of the PCN were examined by SEM (JSM‐6700F, JEOL, Ltd.). The PCN surface was illuminated with a solar simulator (Scientech SF300, Canada) (Greenbrier International, Canada). Thermal conductivity values for the samples were measured with a multifunction rapid thermal conductivity tester (DRE‐III, China). An energy dispersive spectrometer was equipped to detect elements and analyze their contents in samples (EDS, INCA type, British Oxford Instrument Co). The powder XRD patterns were recorded on a D/Max‐2400 X‐ray diffractometer (X pert PRO, Netherlands) using Cu‐Ka radiation, operated at 40 kV and 100 mA from 10° to 80°. XPS analyses were performed using a PHI‐5300ESCA spectrometer (Perkin–Elmer). The adsorption and desorption curves of PCN were determined by the Brunauer–Emmett–Teller (BET, ASAP 2020 V4.03E) specific surface area test method. The optical transmittance and reflectance spectra of the PCN were measured in the range of 200–2500 nm with a Shimadzu UV3600 spectrophotometer attached to an integrating sphere.

## Conflict of Interest

The authors declare no conflict of interest.

## Supporting information

Supporting Information
