# Supplementary material for: Porous Carbon Nanofoam Derived From Pitch as Solar Receiver for Efficient Solar Steam Generation
Source: Glob Chall. 2020 Feb 20;4(5):1900098. doi: 10.1002/gch2.201900098 (PMC7175018; doi:10.1002/gch2.201900098)
Supplement: Supplementary file 1 — Supporting Information [file GCH2-4-1900098-s001.pdf]

Copyright WILEY-VCH Verlag GmbH & Co. KGaA, 69469 Weinheim, Germany, 2020.

Global Challenges
Open Access

Supporting Information

for *Global Challenges,* DOI: 10.1002/gch2.201900098

Porous Carbon Nanofoam Derived From Pitch as Solar Receiver for Efficient Solar Steam Generation

Lihua Chen, Shujing Zhao, Qi-Meige Hasi, Xiaofang Luo, Chuantao Zhang, Hailing Li, and An Li**

Supporting Information

Porous carbon nanofoam derived from pitch as solar receiver for efficient solar steam generation

Lihua Chen[1], Shujing Zhao[1], Qimeige Hasi[1], Xiaofang Luo[1], Chuantao Zhang[1], Hailing Li[1], An Li[2]**

1. Calculation of the energy conversion efficiency

$$\eta = m\, h_{Lv} / C_{opt} q_i$$

where m is the mass flux of steam (the rate of water evaporation under the dark environmentis subtracted), C_{opt} is the optical concentration, q_i is the nominal direct solar irradiation 1 kW m^{-2}, h_{Lv} denotes total enthalpy of liquid-vapor phase change (including sensible heat and phase-change enthalpy), can be calculated as

$$h_{Lv} = \lambda + C\Delta T$$

where λ is latent heat of phase change (correspondence at different temperatures), C is specific heat capacity of water (4.2 kJ kg^{-1} K^{-1}), and ΔT denotes the temperature increase of the water.

2. **Pitch : $CaCO_3$ nanoparticle, SEM image of including 1:4, 1:8, 1:10, 1:12. 1:14 and 1:20.**

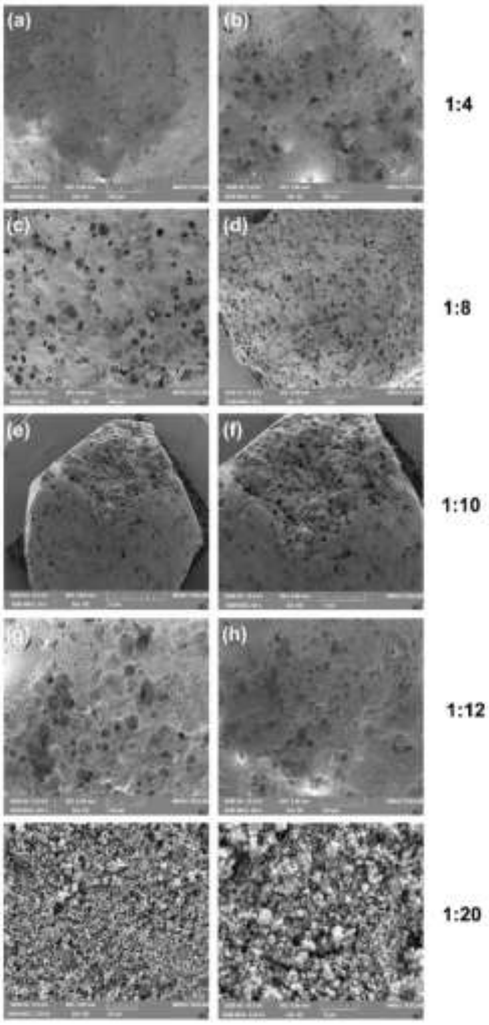

(a)
(b)
1:4
(c)
(d)
1:8
(e)
(f)
1:10
(g)
(h)
1:12
1:20
